# Supplementary material for: Neuromechanical changes in Achilles tendinopathy and the effects of exercise-induced mechanical tendon loading: a protocol for a systematic review
Source: BMJ Open. 2022 Feb 7;12(2):e050186. doi: 10.1136/bmjopen-2021-050186 (PMC8830232; doi:10.1136/bmjopen-2021-050186)
Supplement: Supplementary data [file bmjopen-2021-050186supp002.pdf]

Search strategy step 1 MEDLINE

Search string

("Corticospinal excita*" OR "cortical excita*" OR “spinal excita*” OR "spinal reflex" OR "H-reflex" OR "V-wave" OR "F-wave" OR "neural drive" OR "motor evoked potential*" OR "evoked muscle response*" OR "electromyogra*" OR "activation" OR "co-activation" OR "preactivation" OR "electromechanic*" OR "motor unit*" OR "motor neuron*" OR "length" OR "thickness" OR "cross sectional area" OR "modulus" OR "viscoelasticity" OR "elasticity" OR "strain" OR "stress" OR "stiffness" OR "volume" OR "creep" OR "mechanic*" OR "morphologic*") AND ("tendin*" OR "tenosynovitis" OR "paratenonitis" OR "tendovaginitis" OR "peritendinitis" OR "achillodynia") AND ("Achilles" OR "TendoAchilles" OR "Tendo-Achilles" OR "calcaneal tendon" OR "gastrocnemius" OR "triceps surae" OR "calf muscle*" OR "tibialis anterior")

Keywords

1- Corticospinal excita*.mp
2- Cortical excita*.mp
3- Spinal excita*.mp
4- Spinal reflex.mp
5- H-reflex.mp
6- V-wave.mp
7- F-wave.mp
8- Neural drive.mp
9- Motor evoked potential*.mp
10- Evoked muscle response*.mp
11- Electromyogra*.mp
12- Activation.mp
13- Co-activation.mp
14- Preactivation.mp
15- Electromechanic*.mp
16- Motor unit*.mp
17- Motor neuron*.mp
18- Length.mp
19- Thickness.mp
20- Cross sectional area.mp
21- Modulus.mp
22- Viscoelasticity.mp
23- Elasticity.mp
24- Strain.mp
25- Stress.mp
26- Stiffness.mp
27- Volume.mp

28- Creep.mp
29- Mechanic*.mp
30- Morphologic*.mp
31- 1-30
32- Tendin*.mp
33- Tenosynovitis.mp
34- Paratenonitis.mp
35- Tendovaginitis.mp
36- Peritendinitis.mp
37- Achillodynia.mp
38- 32-37
39- Achilles.mp
40- TendoAchilles.mp
41- Tendo-Achilles.mp
42- Calcaneal tendon.mp
43- Gastrocnemius.mp
44- Triceps surae.mp
45- Calf muscle*.mp
46- Tibialis anterior.mp
47- 39-46
48- 31 AND 38 AND 47

Search strategy step 2 MEDLINE

Search string

("corticospinal excita*" OR "cortical excita*" OR “spinal excita*” OR "spinal reflex" OR "H-reflex" OR "V-wave" OR "F-wave" OR "neural drive" OR "motor evoked potential*" OR "evoked muscle response*" OR "electromyogra*" OR "activation" OR "co-activation" OR "preactivation" OR "electromechanic*" OR "motor unit*" OR "motor neuron*" OR "length" OR "thickness" OR "cross sectional area" OR "modulus" OR "viscoelasticity" OR "elasticity" OR "strain" OR "stress" OR "stiffness" OR "volume" OR "creep" OR "mechanic*" OR "morphologic*") AND ("mechanical load*" OR "Exercise*" OR "physical activity*" OR "eccentric" OR "concentric" OR "isometric" OR "training" OR "strengthening" OR "stretching" OR "vibration" OR "oscillation" OR "plyometric" OR "running" OR "walking") AND ("tendin*" OR "tenosynovitis" OR "paratenonitis" OR "tendovaginitis" OR "peritendinitis" OR "achillodynia") AND ("Achilles" OR "TendoAchilles" OR "Tendo-Achilles" OR "calcaneal tendon" OR "gastrocnemius" OR "triceps surae" OR "calf muscle*" OR "tibialis anterior")

Keywords

1- Corticospinal excita*.mp
2- Cortical excita*.mp
3- Spinal excita*.mp
4- Spinal reflex.mp
5- H-reflex.mp
6- V-wave.mp
7- F-wave.mp
8- Neural drive.mp
9- Motor evoked potential*.mp
10- Evoked muscle response*.mp
11- Electromyogra*.mp
12- Activation.mp
13- Co-activation.mp
14- Preactivation.mp
15- Electromechanic*.mp
16- Motor unit*.mp
17- Motor neuron*.mp
18- Length.mp
19- Thickness.mp
20- Cross sectional area.mp
21- Modulus.mp
22- Viscoelasticity.mp
23- Elasticity.mp
24- Strain.mp

25- Stress.mp
26- Stiffness.mp
27- Volume.mp
28- Creep.mp
29- Mechanic*.mp
30- Morphologic*.mp
31- 1-30
32- Mechanical load*.mp
33- Exercise*.mp
34- Physical activity*.mp
35- Eccentric.mp
36- Concentric.mp
37- Isometric.mp
38- Training.mp
39- Strengthening.mp
40- Stretching.mp
41- Vibration.mp
42- Oscillation.mp
43- Plyometric.mp
44- Running.mp
45- Walking.mp
46- 32-45
47- Tendin*.mp
48- Tenosynovitis.mp
49- Paratenonitis.mp
50- Tendovaginitis.mp
51- Peritendinitis.mp
52- Achillodynia.mp
53- 47-52
54- Achilles.mp
55- TendoAchilles.mp
56- Tendo-Achilles.mp
57- Calcaneal tendon.mp
58- Gastrocnemius.mp
59- Triceps surae.mp
60- Calf muscle*.mp
61- Tibialis anterior.mp
62- 54-61
63- 31 AND 46 AND 53 AND 62
