# Supplementary material for: Neuromechanical changes in Achilles tendinopathy and the effects of exercise-induced mechanical tendon loading: a protocol for a systematic review
Source: BMJ Open. 2022 Feb 7;12(2):e050186. doi: 10.1136/bmjopen-2021-050186 (PMC8830232; doi:10.1136/bmjopen-2021-050186)
Supplement: Author's manuscript [file bmjopen-2021-050186.draft_revisions.pdf]

BMJ Open

BMJ Open is committed to open peer review. As part of this commitment we make the peer review history of every article we publish publicly available.

When an article is published we post the peer reviewers' comments and the authors' responses online. We also post the versions of the paper that were used during peer review. These are the versions that the peer review comments apply to.

The versions of the paper that follow are the versions that were submitted during the peer review process. They are not the versions of record or the final published versions. They should not be cited or distributed as the published version of this manuscript.

BMJ Open is an open access journal and the full, final, typeset and author-corrected version of record of the manuscript is available on our site with no access controls, subscription charges or pay-per-view fees (http://bmjopen.bmj.com).

If you have any questions on BMJ Open's open peer review process please email info.bmjopen@bmj.com

BMJ Open

# Neuromechanical changes in Achilles tendinopathy and the effects of exercise-induced mechanical tendon loading. A protocol for a systematic review.



| | |
|---|---|
| Journal: | *BMJ Open* |
| Manuscript ID | bmjopen-2021-050186 |
| Article Type: | Protocol |
| Date Submitted by the Author: | 12-Feb-2021 |



| | |
|---|---|
| Complete List of Authors: | Contreras-Hernandez, Ignacio; University of Birmingham, School of Sport, Exercise and Rehabilitation Sciences<br>Falla, Deborah; University of Birmingham, School of Sport, Exercise and Rehabilitation Sciences<br>Schneebeli, Alessandro; University of Applied Sciences and Arts of Southern Switzerland,<br>Martinez-Valdes, Eduardo; University of Birmingham, School of Sport, Exercise and Rehabilitation Sciences |



| | |
|---|---|
| Keywords: | SPORTS MEDICINE, REHABILITATION MEDICINE, Foot & ankle < ORTHOPAEDIC & TRAUMA SURGERY |
| | |


SCHOLARONE™
Manuscripts

# Title

***Neuromechanical changes in Achilles tendinopathy and the effects of exercise-induced mechanical tendon loading. A protocol for a systematic review.***

**Authors:** Ignacio Contreras-Hernandez[1], Deborah Falla[1], Alessandro Schneebeli [1 2], Eduardo Martinez-Valdes[1].

*Corresponding author: Eduardo Martinez-Valdes[1]

Email: e.a.martinezvaldes@bham.ac.uk

## Author Affiliations

[1]Centre of Precision Rehabilitation for Spinal Pain (CPR Spine), School of Sport, Exercise and Rehabilitation Sciences, College of Life and Environmental Sciences, University of Birmingham, Birmingham, UK.

[2]Rehabilitation Research Laboratory 2rLab, Department of Business Economics, Health and Social Care, University of Applied Sciences and Arts of Southern Switzerland, Manno/Landquart, Switzerland.

Email: Ignacio Contreras-Hernandez[1] iac921@student.bham.ac.uk – Deborah Falla[1] d.falla@bham.ac.uk – Alessandro Schneebeli[1 2] alessandro.schneebeli@supsi.ch – Eduardo Martinez-Valdes[1] e.a.martinezvaldes@bham.ac.uk

**WORD COUNT MAIN TEXT: 3236 (excluding title page, abstract, references, figures and tables)**

For peer review only

# Abstract

**Introduction:** Achilles tendinopathy is a debilitating overuse injury characterised by pain, impaired functional performance, morpho-mechanical changes to the Achilles tendon, and triceps surae neuromuscular alterations. Loading-based exercise has become the principal nonsurgical choice for the treatment of Achilles tendinopathy, however, mechanistic evidence by which loading-based treatment may help to resolve tendon pain remains unclear. This systematic review aims to summarize the evidence of the neuromechanical changes produced by Achilles tendinopathy and by exercise-induced mechanical loading.

**Methods and analysis:** This systematic review protocol was informed and reported in accordance with the Preferred Reporting Items for Systematic Review and Meta-Analysis (PRISMA-P) and the Cochrane Handbook for Systematic Reviews of Interventions. Pubmed, MEDLINE, EMBASE, CINAHL Plus, Web of Science, and ZETOC electronic databases will be searched from inception to 31 February 2021. Additionally, grey literature and key journals will be reviewed. Risk of bias will be determined independently by two reviewers using RoB2 and ROBINS-I tools according to Cochrane recommendations. Quality of the cumulative evidence will be assessed with the Grading of Recommendations, Assessment, Development, and Evaluation (GRADE) guidelines. If homogeneity exists between groups of studies, a random-effects meta-analysis will be conducted. If not, results will be synthesised narratively.

**Ethics and dissemination:** Our findings will be disseminated through publication in a peer-review journal and presented at conferences. No ethical approval was required.


**Prospero registration number:** CRD42021231933

**Keywords:** Achilles tendinopathy; neuromechanical properties, neuromuscular features, mechanical loading.

**WORD COUNT: 233**

For peer review only

# Article Summary

## Strengths and limitations of this study

- This will be the first systematic review to synthesise evidence of neuromechanical changes produced by Achilles tendinopathy.
- This will also be the first systematic review examining the effect of exercise-induced mechanical loading protocols on neuromechanical changes observed in individuals with Achilles tendinopathy.
- In accordance with the Grading of Recommendations, Assessment, Development, and Evaluation framework, the inclusion of observational studies might reduce the quality of evidence.

For peer review only

# INTRODUCTION

Tendinopathy describes a spectrum of pathological changes to the tendon, leading to pain and reduced function. Tendinopathy is characterised by abnormalities in the microstructure, composition, and cellularity of the tendon.[1] The essence of tendinopathy is a failed healing response, with degeneration and disorganized proliferation of tenocytes, disruption of collagen fibres, and subsequent increase in non-collagenous matrix.[2]

Achilles tendinopathy (AT) is a debilitating overuse injury which causes considerable morbidity and functional impairment among the athletic and general population.[3 4] Morphological comparisons of tendinopathic and healthy tendons have demonstrated a larger cross-sectional area (CSA) for the degenerated Achilles tendon.[5] It is believed that this increase is due to an accumulation of water and increased ground substance as a result of the pathology.[6] In addition, changes to the tendon's stiffness has been reported.[5] Typically, a thicker tendon is considered mechanically stronger due to its ability to dissipate high stresses (force/area) across the tendon and yield lower strain energy.[5] However, degenerated Achilles tendon has lower stiffness and Young's modulus compared with healthy tendons.[5]

Tendons require the ability to withstand, store, and then deliver substantial force to perform day-to-day activities.[1] During sports-related activities, where the repetition and speed of the loading are drastically increased, the mechanical force placed on the tendon becomes substantially amplified.[7] Therefore, a decrease in tendon stiffness will cause the muscle fascicles to shorten more to account for the increased compliance of the tendon.[5] This may limit the muscle's ability to function within the force-length curve's optimal region, thereby affecting movement economy.[5] In addition to the morpho-mechanical changes induced by AT, several neuromechanical (coupling between neural information processing and mechanical behavior[8]) adaptations have been reported in individuals with AT.[9] Neuromechanical adaptations include how the mechanical system may offload a task that the neural system needs to accomplish. The size, shape, structural properties, and even the physics of the medium may determine how the neural system functions to control movement, and how processing of sensory information may limit the range or rates of movements that are feasible.[8] One of these neuromechanical adaptations is the electromechanical delay

For peer review only

(EMD), which is defined as the time lag between muscle activation and the mechanical force produced, which dictates the muscle-tendon unit's temporal efficiency.[9] Longer EMD has recently been reported for the affected side compared with the non-affected side in individuals with Achilles tendinosis, indicating a compromised triceps surae musculotendinous unit temporal efficiency (ability to transmit force from the muscle to the tendon as quickly as possible).[9] A theory supporting changes in neuromechanical function with AT is that the CNS (central nervous system) can adapt to the muscle-tendon unit's impaired temporal efficiency modifying the evoked spinal and supraspinal responses.[9] These adaptations indicated an enhanced neural drive in descending corticospinal pathways, elevated motoneuron excitability, and/or alterations in presynaptic inhibition.[10]

Once symptoms develop, ensuing movement dysfunction may contribute to the chronicity of symptoms.[11] Pain in the Achilles tendon causes widespread motor inhibition in the affected region, evidenced by the lower electrical activity of the agonist, synergist, and antagonist muscles.[12]. Individuals with tendinopathy also tend to use movement patterns that place excessive or abnormal load on their tendons; the faulty movement may represent either a root cause or a reason for chronicity or slow resolution.[11] This may be attributed to a protective mechanism that prevents further injury or even tendon rupture.[13]

Research on the treatment of AT is somewhat scarce despite the prevalence.[14] Over the past decade, loading-based treatment in the form of eccentric training (exercises where tendon lengthening during active contractions is emphasized) has become the main non-surgical choice of treatment for AT,[15] although there is no convincing evidence showing that this form of exercise is the most effective for AT. A recent systematic review concluded that there is little clinical or mechanistic evidence supporting the use of isolated eccentric exercises alone.[16] Well-conducted studies comparing different loading programs are largely lacking.[16] The purpose of exercise is to provide mechanical loading to the tendon in order to promote remodeling, decrease pain, and improve calf-muscle endurance and strength.[17 18] It seems that loading itself yields positive clinical, structural, and biochemical effects with respect to tendinopathy.[17 19-22] However, the successful management of AT remains challenging, possibly due to a lack of knowledge about the

For peer review only

effect of loading parameters i.e., load progression, load magnitude, frequency (sets and repetitions), and restitution between treatment sessions.[14]

There are several systematic reviews regarding the effects of exercise in individuals with AT. However, most are focused on pain and function and usually only use self-reported outcome measures as a main outcome. Self-reported outcomes have high variability among the population; thus, the conclusion of these systematic reviews may be partially biased. Although two recent systematic reviews explored the effects of mechanical loading on the Achilles tendon morphological properties in individuals with mid-portion AT,[23 24] there are currently no systematic reviews examining 1) Triceps surae-Achilles tendon complex neuromechanics in individuals with AT 2) The effect of exercise-induced tendon loading on neuromechanical changes induced by AT to this region. Thus, the aim of this systematic review is to synthesise the current literature to determine the neuromechanical changes that occur in individuals with AT and the effects of exercised mechanical tendon loading on these properties.

For peer review only

# METHODS

This systematic review protocol has been developed following the Cochrane Handbook for Systematic Reviews of Interventions and the Preferred Reporting Items for Systematic Review and Meta-analysis Protocols (PRISMA-P) 2015 checklist (supplementary file 1).[25-27] This protocol has been registered with the International Prospective Register of Systematic Reviews (PROSPERO) on 26-01-2021 (Registration number: CRD42021231933).

## Eligibility criteria

The PICOS framework (Population, Intervention, Comparison, Outcomes, and Study Design) has been used to define the eligibility criteria for the inclusion and exclusion of studies in this systematic review.[26 28] However, due to the characteristics of this research, we will use "Indicator" in the same category of "Intervention" as it has been used previously.[29]

## Population

The population of interest is adults (aged 18 to 65 years) with mid-portion or insertional Achilles tendinopathy and adults who are pain-free as a control group. In order to avoid excluding relevant articles identified during the scoping search, participants with bilateral Achilles tendinopathy will also be included. There will be no restrictions in terms of gender and/or ethnicity. Studies that include individuals with Achilles tendinopathy who have been diagnosed with an underlying medical pathology or disorder (e.g. systemic inflammatory conditions, cardiovascular diseases, neurological disorders) and/or history of Achilles tendon surgery will be excluded.

## Intervention/Indicator

Eligible studies will be those which include the use of any electrophysiological technique (e.g., surface electromyography, intramuscular electromyography, high-density surface electromyography, transcranial magnetic stimulation, cervicomedullary magnetic stimulation, or peripheral nerve stimulation) to determine neuromuscular, spinal, subcortical, or supraspinal changes in people with AT or produced by exercise-induced mechanical loading in individuals with this condition. Additionally, eligible studies will be those which include the use of

For peer review only

ultrasonographic or Magnetic Resonance Imaging (MRI) to measure the morphological or mechanical properties of the Achilles tendon in people with AT as well as changes by exercise-induced mechanical loading in individuals with this disorder.

## Comparison

Studies must include a comparison of the neuromuscular (leg muscles) or morpho-mechanical properties of the Achilles tendon in individuals with and without AT. Likewise, studies assessing the effects of exercise-induced mechanical tendon loading should include a comparison of the neuromuscular or morpho-mechanical properties of the Achilles tendon in individuals with and without AT. The comparison could be within-groups (e.g., symptomatic and asymptomatic lower limb) or between-groups (e.g., Achilles tendinopathy group and control group).

## Outcomes

Neuromuscular properties of the triceps surae muscle and morpho-mechanical properties of the AT will be considered. We will include studies assessing the amplitude and timing of EMG activity of the gastrocnemius-soleus, tibialis anterior and gastrocnemius-soleus co-activation, gastrocnemius-soleus motor evoked potentials (MEPs) obtained from transcranial and cervicomedullary magnetic stimulation (TMS and CMEPs), H-reflex, F-wave or V-wave obtained by peripheral nerve stimulation (tibial nerve) and motor unit data obtained from intramuscular and/or high-density surface EMG recordings. Morpho-mechanical properties such as length, thickness, cross-sectional area, volume, stiffness, modulus, creep, elasticity, strain, and stress of the Achilles tendon will be also included in the present review. Only studies that measure any of these neuromechanical properties quantitatively will be included.

## Study design

Based on scoping searches, randomised controlled trials and non-randomised controlled trials (i.e., cohort, cross-sectional, and cohort studies) will be considered to properly address the review´s research question. Non-original literature (e.g., systematic and narrative reviews) or other types of studies will be excluded and reported on the PRISMA flow diagram.

For peer review only

## Information sources

The following electronic databases will be used from inception to 31 February 2021: Pubmed, MEDLINE (Ovid Interface), EMBASE (Ovid Interface), CINAHL Plus (EBSCO Interface), Web of Science (WOS; Clarivate Analytics) and ZETOC. Specific research strategies have been designed considering medical subject heading (MESH) terms to improve search results. Moreover, hand searching of key journals will be conducted, including Journal of Applied Physiology, Journal of Orthopaedic & Sports Physical Therapy, Journal of Electrophysiology and Kinesiology, Journal of Biomechanics, Clinical Biomechanics, British Journal of Sports Medicine, Medicine and Science in Sports and Exercise, Journal of Science and Medicine in Sports, and Isokinetic and Exercise Science. The eligibility of the manuscripts found in hand searching will be defined using the PICOS framework. Additionally, relevant authors in the field will be contacted to identify unpublished articles in preparation. In order to minimise risk of bias publication, grey literature will be also included, and searches will be conducted using the British national bibliography for report literature (BNBRL), ProQuest Dissertations & These Global, OpenGrey database and EThOs. Reference lists of included studies and relevant systematic reviews will be checked for any further studies, accordingly with the MECIR standards.[30]

## Search strategy

The lead author (IC) will conduct the search strategy. There will be no restrictions in terms of date, format, design, region, or language. A search strategy example for MEDLINE (Ovid Interface) database is reported in online supplementary file 2 and includes MESH terms and keyword searching to ensure maximal retrieval.[31] The specific search terms will be modified to reflect differences in keywords and syntax between databases, but search strategy consistency will be guaranteed.

## Data management

Literature search results, including citation and abstract of potentially eligible studies, will be imported into EndNote X9 (Clarivate Analytics PCL) reference manage software by one reviewer (IC), allowing the identification and removal of any duplicates before the screening process. Abstracts and full-texts of potentially eligible studies will be saved in an individual folder for each reviewer (IC, AS) and eligible studies will be retrieved and stored in EndNote X9. To

For peer review only

effectively accomplish the screening process, forms that have been developed to reflect the inclusion and exclusion criteria will be used.

## Study selection

Before the screening process, screening forms will be tested by two reviewers (IC, AS) in a small number of articles to ensure their effectiveness. The screening process will then start with the assessment of titles and abstracts of identified studies by two reviewers (IC, AS), and they will subcategorise them into definitely eligible, definitely ineligible, or doubtful.[32] In the event of disagreement, reviewers will first attempt to resolve through discussion; however, if consensus cannot be reached, a third reviewer (EMV) will mediate the process. Then, the reviewers will perform full-text screening of potentially eligible studies independently. Similarly, if no consensus is possible, a third reviewer will support the process. The agreement between the reviewers during both screening stages will be determined using the kappa statistic, and the PRISMA flow diagram will be used to summarise the study selection process.[26]

## Data collection process

The data collection process will begin developing a standardised form based on the Cochrane data extraction template, objectives of the systematic review, and inclusion criteria as a guide. A standardised form will be piloted a priori on a sub-group of studies. IC will extract data, and AS will check the accuracy of this process. Any discrepancies will be discussed between the two reviewers; however, the third reviewer (EMV) will determine which data are relevant if no agreement is achieved. Authors of the primary studies will be contacted if any critical information that needs to be extracted is missing. If multiple publications of the same study exist, they will be collated, the primary authors contacted for further clarification and the duplicates removed. Likewise, if potentially eligible studies appear to use the same data during the data collection process, the primary authors will be contacted, and a specific report will be selected. The decision of the selected report will be justified.

For peer review only

## Data items

Data items to be extracted include study general information, participants´ description, measurement methods, and outcome measures. These items are presented in Table 1. Data will be extracted from the control and Achilles tendinopathy group. If any of the eligible studies include more than two groups, data will be extracted only from the control group and the one that meets the eligibility criteria.

**Table 1.** Characteristics of included studies

**Date of data extraction:**

| Information about data | Data extracted |
|---|---|
| General study information | Title<br>Authors<br>Year of publication |
| Study Methodology | Study design<br>Study setting (including country)<br>Sample size<br>Individuals' characteristics (age, gender; for both Achilles tendinopathy and control group)<br>Diagnostic confirmation (clinical evaluation, ultrasound/MRI assessment, use of questionnaires, etc)<br>Achilles tendinopathy group characteristics (location, side, pain intensity, duration of symptoms, etc)<br>Type of instrument used to measure the neuromuscular properties<br>Type of instrument used to determine the morpho-mechanical properties (Ultrasonography, MRI, or dynamometer)<br>Type of exercise-induced mechanical tendon loading protocol applied |
| Outcome | The neuromuscular properties include the amplitude and timing of EMG activity of the gastrocnemius-soleus, tibialis anterior and gastrocnemius-soleus co-activation, gastrocnemius-soleus MEPs, H-reflex, F-wave, and motor unit data.<br>The morpho-mechanical properties of the Achilles tendon include length, thickness, cross-sectional area, volume, stiffness, modulus, creep, elasticity, strain, and stress.<br>The comparison could be within-groups (e.g. affected versus non-affected side) or between-groups (e.g. Achilles tendinopathy group versus control group). |

**Risk of bias**

The risk of bias will be determined independently by two reviewers (ICH and AS) using the RoB 2 and ROBINS-I tools according with Cochrane recommendations.[25] The RoB 2 is a tool to determine the risk of bias in randomised trials and includes the following domains: bias arising from the randomisation process, bias due to deviations from intended interventions, bias due to missing outcome data, bias in measurement of the outcome, bias in selection of the reported result, and overall bias.[33] The domains were selected to address all important mechanisms by which bias can be introduced into the results of a trial, based on a combination of empirical evidence and theoretical considerations.[33] Each domain is required, and no additional domains should be added. For each domain, the tool comprises a series of "signalling questions", a judgement about risk of bias for the domain, free text boxes to justify responses to the signalling questions and risk-of-bias judgement, and an option to predict (and explain) the likely direction of bias.[25] Signalling questions aim to elicit information relevant to an assessment of risk of bias.[33] The questions seek to be reasonably factual in nature.[33] The response options are "yes," "probably yes," "probably no," "no," and "no information".[33] Bases on these responses, the options for a domain-level risk-of-bias judgement are "Low", "Some concerns", and "High" risk of bias.[33]

The ROBINS-I will be used to determine the risk of bias in non-randomised studies of interventions and include the following domains: bias due to confounding, bias in selection of participants into the study, bias in classification of interventions, bias due to deviations from intended interventions, bias due to missing data, bias in measurement of outcomes, and bias in the selection of the reported result.[34] Each domain is mandatory, and no additional domain should be added. The tool comprises, for each domain a series of "signalling questions", a judgement about risk of bias for the domain, free text boxes to justify responses to the signalling questions and risk-of-bias judgements, and an option to predict (and explain) the likely direction of bias.[25] The signalling questions aim to elicit information relevant to the risk-of-bias judgement for the domain, and work in the same way as for RoB 2.[25] The response options are "yes," "probably yes," "probably no," "no," and "no information".[34] Bases on these responses, the options for a domain-level risk-of-bias judgement are "Low", "Moderate", "Serious" or "Critical" risk of bias, with an additional option of "No information".[34]

Disagreements between the reviewers regarding the risk of bias in a study will be resolved by discussion, with the involvement of a third review author (EMV) if necessary.

For peer review only - http://bmjopen.bmj.com/site/about/guidelines.xhtml

## Data synthesis

A meta-analysis will be considered if outcomes and methodology of the selected studies are homogenous. If possible, the two reviewers (IC, AS) will independently group studies that are more homogeneous using the following characteristics:

► Parameter used to determine neuromuscular properties of the Triceps Surae

► Parameter used to measure morpho-mechanical properties of the Achilles tendon

► Type of exercised-induced mechanical tendon loading protocol applied (eg., concentric, eccentric, isometric, plyometric, etc.)

Disagreement between the reviewers will be resolved by discussion, but if no agreement is possible, a third reviewer (EMV) will mediate the process.

Whether clinical or methodological homogeneity across studies investigating the same outcome domain is sufficient, statistical heterogeneity will be performed. The amount of inconsistency among studies will be assessed using the $I^2$ statistic.[35] As in previous reviews, the grouping of studies will be eligible for meta-analysis if an $I^2$ value of <50% (low heterogeneity) is determined.[36] Then, a random-effects meta-analysis will be performed for each subgroup following the recommendations of Deeks, et al, 2019.[37] Extracted data will be converted into a common rubric, which most likely be odds ratios with 95% confidence intervals since we are dealing mostly with binary data.[38] If the data are not sufficiently homogenous, the results of the considered outcomes will be described using the vote-counting procedure (direction of difference or no difference) and a narrative synthesis will be developed.[39] The narrative synthesis will be conducted following the recommendations of Popay, et al., 2006.[38]

## Confidence in cumulative evidence

Data pooled quality (certainty) will be assessed using the Grading of Recommendations Assessment, Development, and Evaluation (GRADE) approach. [40 41] This process include 5 steps described by Goldet and Howick, (2013)[42] and the final quality of evidence will be presented as 'High', 'Moderate', 'Low' or 'Very Low'. The certainty of evidence for each outcome across studies can be decreased by risk of bias, inconsistency, indirectness, imprecision and publication bias.[43] Conversely, certainty of evidence can be increased by large effect size, dose-response gradient, and plausible confounding biases that underestimate the effect size.[43] Finally, recommendations

For peer review only

for the interpretation of the evidence quality will be given, following the criteria of Guyatt et al., (2008).[44]

## Patient and public involvement

The topic of this systematic review protocol was not discussed at our established patient and public involvement meetings (PPI), due to COVID-19 pandemic. Patients will not be involved in the analysis and data collection of this project, but our results will be presented at PPI meetings at the University of Birmingham in the future.

## Ethics and dissemination of results

Ethical approval is not required for this review, as it will only involve the collation of previously published data. Our findings will be disseminated through publication in a peer-review journal and presented at national and/or international conferences.

**Author Contributions:** IC and EMV are responsible for the conception of the research question and development of the protocol. IC wrote the first draft of the protocol with guidance from EMV and DF. IC and AS will be the first and second reviewers, respectively. EMV will be the third reviewer. All drafts were revised and reviewed by all the authors before the final approval of the last version of the manuscript.

**Funding:** The authors have not declared a specific grant for this research from any funding agency in the public, commercial or not-for-profit sectors.

**Competing interests:** The authors have no competing interests to declare.

**Patients consent for publication:** Not required.

**Data availability statement:** This work is a systematic review protocol, and as such all data is derived from previously published work.

**Acknowledgements:** Not applicable.

For peer review only

## REFERENCES


1. Millar NL, Silbernagel KG, Thorborg K, et al. Tendinopathy. *Nat Rev Dis Primers* 2021;7(1):1.
2. Longo UG, Ronga M, Maffulli N. Achilles tendinopathy. *Sports Med Arthrosc Rev* 2009;17(2):112-26.
3. Kvist M. Achilles tendon injuries in athletes. *Sports Med* 1994;18(3):173-201.
4. Rolf C, Movin T. Etiology, histopathology, and outcome of surgery in achillodynia. *Foot Ankle Int* 1997;18(9):565-9.
5. Arya S, Kulig K. Tendinopathy alters mechanical and material properties of the Achilles tendon. *J Appl Physiol (1985)* 2010;108(3):670-5.
6. Maffulli N, Moller HD, Evans CH. Tendon healing: can it be optimised? *Br J Sports Med* 2002;36(5):315-6.
7. Lin TW, Cardenas L, Soslowsky LJ. Biomechanics of tendon injury and repair. *Journal of Biomechanics* 2004;37(6):865-77.
8. Nishikawa K, Biewener AA, Aerts P, et al. Neuromechanics: an integrative approach for understanding motor control. *Integr Comp Biol* 2007;47(1):16-54.
9. Chang YJ, Kulig K. The neuromechanical adaptations to Achilles tendinosis. *J Physiol* 2015;593(15):3373-87.
10. Aagaard P, Simonsen EB, Andersen JL, et al. Neural adaptation to resistance training: changes in evoked V-wave and H-reflex responses. *J Appl Physiol (1985)* 2002;92(6):2309-18.
11. Scott A, Backman LJ, Speed C. Tendinopathy: Update on Pathophysiology. *J Orthop Sports Phys Ther* 2015;45(11):833-41.
12. Henriksen M, Aaboe J, Graven-Nielsen T, et al. Motor responses to experimental Achilles tendon pain. *Br J Sports Med* 2011;45(5):393-8.
13. Lund JP, Donga R, Widmer CG, et al. The pain-adaptation model: a discussion of the relationship between chronic musculoskeletal pain and motor activity. *Can J Physiol Pharmacol* 1991;69(5):683-94.
14. Beyer R, Kongsgaard M, Hougs Kjær B, et al. Heavy Slow Resistance Versus Eccentric Training as Treatment for Achilles Tendinopathy: A Randomized Controlled Trial. *Am J Sports Med* 2015;43(7):1704-11.
15. Alfredson H, Pietilä T, Jonsson P, et al. Heavy-load eccentric calf muscle training for the treatment of chronic Achilles tendinosis. *Am J Sports Med* 1998;26(3):360-6.
16. Malliaras P, Barton CJ, Reeves ND, et al. Achilles and patellar tendinopathy loading programmes : a systematic review comparing clinical outcomes and identifying potential mechanisms for effectiveness. *Sports Med* 2013;43(4):267-86.
17. Silbernagel KG, Thomeé R, Eriksson BI, et al. Continued sports activity, using a pain-monitoring model, during rehabilitation in patients with Achilles tendinopathy: a randomized controlled study. *Am J Sports Med* 2007;35(6):897-906.
18. Silbernagel KG, Crossley KM. A Proposed Return-to-Sport Program for Patients With Midportion Achilles Tendinopathy: Rationale and Implementation. *J Orthop Sports Phys Ther* 2015;45(11):876-86.
19. Alfredson H, Lorentzon R. Chronic Achilles tendinosis: recommendations for treatment and prevention. *Sports Med* 2000;29(2):135-46.
20. Kongsgaard M, Kovanen V, Aagaard P, et al. Corticosteroid injections, eccentric decline squat training and heavy slow resistance training in patellar tendinopathy. *Scand J Med Sci Sports* 2009;19(6):790-802.


For peer review only

21. Kongsgaard M, Qvortrup K, Larsen J, et al. Fibril morphology and tendon mechanical properties in patellar tendinopathy: effects of heavy slow resistance training. *Am J Sports Med* 2010;38(4):749-56.
22. Mafi N, Lorentzon R, Alfredson H. Superior short-term results with eccentric calf muscle training compared to concentric training in a randomized prospective multicenter study on patients with chronic Achilles tendinosis. *Knee Surg Sports Traumatol Arthrosc* 2001;9(1):42-7.
23. Merza E, Pearson S, Lichtwark G, et al. Immediate and long-term effects of mechanical loading on Achilles tendon volume: A systematic review and meta-analysis. *Journal of Biomechanics* 2021:110289.
24. Färnqvist K, Pearson S, Malliaras P. Adaptation of Tendon Structure and Function in Tendinopathy With Exercise and Its Relationship to Clinical Outcome. *J Sport Rehabil* 2020;29(1):107-15.
25. Higgins JPT TJ, Chandler J, et al. (editors). Cochrane Handbook for Systematic Reviews of Interventions version 6.0 (updated July 2019): Cochrane, 2019.
26. Shamseer L, Moher D, Clarke M, et al. Preferred reporting items for systematic review and meta-analysis protocols (PRISMA-P) 2015: elaboration and explanation. *BMJ : British Medical Journal* 2015;349:g7647.
27. Moher D, Shamseer L, Clarke M, et al. Preferred reporting items for systematic review and meta-analysis protocols (PRISMA-P) 2015 statement. *Systematic Reviews* 2015;4(1):1.
28. Smith V, Devane D, Begley CM, et al. Methodology in conducting a systematic review of systematic reviews of healthcare interventions. *BMC Medical Research Methodology* 2011;11(1):15.
29. Arvanitidis M, Falla D, Sanderson A, et al. Does pain influence force steadiness? A protocol for a systematic review. *BMJ Open* 2021;11(1):e042525.
30. Higgins JPT LT, Chandler J, Tovey D, Churchill R. Methodological Expectations of Cochrane Intervention Reviews. Cochrane: London, 2016.
31. Jenuwine ES, Floyd JA. Comparison of Medical Subject Headings and text-word searches in MEDLINE to retrieve studies on sleep in healthy individuals. *J Med Libr Assoc* 2004;92(3):349-53.
32. Lefebvre C GJ, Briscoe S, et al. Chapter 4: Searching for and selecting studies. In: Higgins JPT TJ, Chandler J, et al. , ed. Cochrane Handbook for Systematic Reviews of Interventions version 60 (updated July 2019): Cochrane, 2019.
33. Sterne JAC, Savović J, Page MJ, et al. RoB 2: a revised tool for assessing risk of bias in randomised trials. *BMJ* 2019;366:l4898.
34. Sterne JA, Hernán MA, Reeves BC, et al. ROBINS-I: a tool for assessing risk of bias in non-randomised studies of interventions. *BMJ* 2016;355:i4919.
35. Higgins JP, Thompson SG. Quantifying heterogeneity in a meta-analysis. *Stat Med* 2002;21(11):1539-58.
36. Heneghan NR, Smith R, Rushton A. Thoracic dysfunction in whiplash-associated disorders: a systematic review and meta-analysis protocol. *Syst Rev* 2016;5:26.
37. Deeks JJ HJ, Altman DG. Chapter 10: analysing data and undertaking meta-analysis. In: Higgins JPT TJ, Chandler J, et al., ed. Cochrane Handbook for systematic reviews of interventions version 60 (updated July 2019): Cochrane, 2019.
38. Popay J RH, Snowden A. Guidance on the conduct of narrative synthesis in systematic reviews: a product from the ESRC methods programme. Lancaster, UK: Lancaster University, 2006.
39. McKenzie JE BS. Chapter 12: Synthesizing and presenting findings using other methods. In: Higgins JPT TJ, Chandler J,, ed. Cochrane Handbook for systematic reviews of interventions. London: Cochrane, 2008.
40. Guyatt G, Oxman AD, Akl EA, et al. GRADE guidelines: 1. Introduction-GRADE evidence profiles and summary of findings tables. *J Clin Epidemiol* 2011;64(4):383-94.

For peer review only

41. Schünemann H BJ, Guyatt G, eds. Handbook for grading the quality of evidence and the strength of recommendations using the GRADE approach2013.
42. Goldet G, Howick J. Understanding GRADE: an introduction. *J Evid Based Med* 2013;6(1):50-54.
43. Balshem H, Helfand M, Schünemann HJ, et al. GRADE guidelines: 3. Rating the quality of evidence. *J Clin Epidemiol* 2011;64(4):401-6.
44. Guyatt GH, Oxman AD, Vist GE, et al. GRADE: an emerging consensus on rating quality of evidence and strength of recommendations. *BMJ* 2008;336(7650):924-26.

For peer review only

**Search strategy MEDLINE**

1- Corticospinal excita*.mp
2- Cortical excita*.mp
3- Spinal excita*.mp
4- Spinal reflex.mp
5- H-reflex.mp
6- V-wave.mp
7- F-wave.mp
8- Neural drive.mp
9- Motor evoked potential*.mp
10- Evoked muscle response*.mp
11- Electromyogra*.mp
12- Activation.mp
13- Co-activation.mp
14- Preactivation.mp
15- Electromechanic*.mp
16- Motor unit*.mp
17- Motor neuron*.mp
18- Length.mp
19- Thickness.mp
20- Cross-sectional area.mp
21- Modulus.mp
22- Viscoelasticity.mp
23- Elasticity.mp
24- Strain.mp
25- Stress.mp
26- Stiffness.mp
27- Volume.mp
28- Creep.mp
29- Mechanic*.mp
30- Morphologic*.mp
31- 1-30
32- Mechanical load*.mp
33- Exercise*.mp
34- Physical activity*.mp
35- Eccentric.mp
36- Concentric.mp
37- Isometric.mp
38- Training.mp
39- Strenghthening.mp

For peer review only

40- Stretching.mp
41- Vibration.mp
42- Oscillation.mp
43- Plyometric.mp
44- Running.mp
45- Walking.mp
46- 32-45
47- Tendin*.mp
48- Tenosynovitis.mp
49- Paratenonitis.mp
50- Tendovaginitis.mp
51- Peritendinitis.mp
52- Achillodynia.mp
53- 47-52
54- Achilles.mp
55- TendoAchilles.mp
56- Tendo-Achilles.mp
57- Calcaneal tendon.mp
58- Gastrocnemius.mp
59- Triceps surae.mp
60- Calf muscle*.mp
61- Tibialis anterior.mp
62- 54-61
63- 31 AND 46 AND 53 AND 62

For peer review only

**PRISMA-P (Preferred Reporting Items for Systematic Review and Meta-Analysis Protocols) 2015 checklist: recommended items to address in a systematic review protocol***

| Section and topic | Items N° | Checklist item | Page N° |
|---|---|---|---|
| **ADMINISTRATIVE INFORMATION** | | | |
| Title: | | | |
| Identification | 1a | Identify the report as a protocol of a systematic review | 1 |
| Update | 1b | If the protocol is for an update of a previous systematic review, identify as such | N/A |
| Registration | 2 | If registered, provide the name of the registry (such as PROSPERO) and registration number | 2,7 |
| Authors: | | | |
| Contact | 3a | Provide name, institutional affiliation, e-mail address of all protocol authors; provide physical mailing address of corresponding author. | 1 |
| Contributions | 3b | Describe contributions of protocol authors and identify the guarantor of the review | 14 |
| Amendments | 4 | If the protocol represents an amendment of a previously completed or published protocol, identify as such and list changes; otherwise, state plan for documenting important protocol amendments | N/A |
| Support: | | | |
| Sources | 5a | Indicate sources of financial or other support for the review | 14 |
| Sponsor | 5b | Provide name for the review funder and/or sponsor | N/A |
| Role of sponsor or funder | 5c | Describe role of funder(s), sponsor(s), and/or institution(s), if any, in developing the protocol | N/A |
| **INTRODUCTION** | | | |
| Rationale | 6 | Describe the rationale for the review in the context of what is already known | 4-6 |
| Objectives | 7 | Provide an explicit statement of the question(s) the review will address with reference to participants, interventions, comparators, and outcomes (PICO) | 6 |
| **METHODS** | | | |
| Eligibility criteria | 8 | Specify the study characteristics (such as PICO, study design, setting, time frame) and report characteristics (such as years considered, language, publication status) to be used as criteria for eligibility for the review | 7,8 |
| Information sources | 9 | Describe all intended information sources (such as electronic databases, contact with study authors, trial registers or other grey literature sources) with planned dates of coverage | 9 |
| Search strategy | 10 | Present draft of search strategy to be used for at least one electronic database, including planned limits, such that it could be repeated | 9/Supplementary file 2 |

For peer review only

| | | | |
|---|---|---|---|
| Study records: | | | |
| Data management | 11a | Describe the mechanism(s) that will be used to manage records and data throughout the review | 9,10 |
| Selection process | 11b | State the process that will be used for selecting studies (such as two independents reviewers) through each phase of the review (that is, screening, eligibility, and inclusion in meta-analysis) | 10 |
| Data collection process | 11c | Describe planned methods of extracting data from reports (such as piloting forms, done independently, in duplicate) any processes for obtaining and confirming data from investigators | 10 |
| Data items | 12 | List and define all variables from which data will be sought (such as PICO items, funding sources), any-pre-planned data assumptions and simplifications | 11/Table 1 |
| Outcomes and prioritization | 13 | List and define all outcomes for which data will be sought, including prioritization of main and additional outcomes, with rationale | 11/Table 1 |
| Risk of bias in individual studies | 14 | Describe anticipated methods for assessing risk of bias of individual studies, including whether this will be done at the outcome or study level, or both; state how this information will be used in data synthesis | 12 |
| Data synthesis | 15a | Describe criteria under which study data will be quantitatively synthesised | 13 |
| | 15b | If data are appropriate for quantitative synthesis, describe planned summary measures, methods of handling data and methods of combining data from studies, including any planned exploration of consistency (such as $I^2$, Kendall's $\tau$) | 13 |
| | 15c | Describe any proposed additional analysis (such as sensitivity or subgroup analyses, meta-regression) | 13 |
| | 15d | If quantitative synthesis is not appropriate, describe the type of summary planned | 13 |
| Meta-bias(es) | 16 | Specify any planned assessment of meta-bias(es) (such as publication bias across studies, selective reporting within studies) | 13 |
| Confidence in cumulative evidence | 17 | Describe how the strength of the body of evidence will be assessed (such as GRADE) | 13,14 |

***It is strongly recommended that this checklist be read in conjunction with the PRISMA-P Explanation and Elaboration for important clarification on the items. Amendments to a review protocol should be tracked and dated. The copyright for PRISMA-P (including checklist) is held by the PRISMA-P Group and is distributed under a Creative Commons Attribution License 4.0.**

From: Shamseer L, Moher D, Clarke M, Ghersi D, Liberati A, Petticrew M, Shekelle P, Stewart L. PRISMA-P Group. Preferred reported items for systematic review and meta-analysis protocols (PRISMA-P) 2015: elaboration and explanations. BMJ. 2015 Jan 2; 349:g7647

For peer review only

BMJ Open

# Neuromechanical changes in Achilles tendinopathy and the effects of exercise-induced mechanical tendon loading. A protocol for a systematic review.


| | |
|---|---|
| Journal: | *BMJ Open* |
| Manuscript ID | bmjopen-2021-050186.R1 |
| Article Type: | Protocol |
| Date Submitted by the Author: | 13-Sep-2021 |
| Complete List of Authors: | Contreras-Hernandez, Ignacio; University of Birmingham, School of Sport, Exercise and Rehabilitation Sciences<br>Falla, Deborah; University of Birmingham, School of Sport, Exercise and Rehabilitation Sciences<br>Schneebeli, Alessandro; University of Applied Sciences and Arts of Southern Switzerland,<br>Martinez-Valdes, Eduardo; University of Birmingham, School of Sport, Exercise and Rehabilitation Sciences |
| <b>Primary Subject Heading</b>: | Sports and exercise medicine |
| Secondary Subject Heading: | Rehabilitation medicine |
| Keywords: | SPORTS MEDICINE, REHABILITATION MEDICINE, Foot & ankle < ORTHOPAEDIC & TRAUMA SURGERY |
| | |


SCHOLARONE™
Manuscripts

# Title

***Neuromechanical changes in Achilles tendinopathy and the effects of exercise-induced mechanical tendon loading. A protocol for a systematic review.***

**Authors:** Ignacio Contreras-Hernandez[1], Deborah Falla[1], Alessandro Schneebeli [1 2], Eduardo Martinez-Valdes[1].

*Corresponding author: Eduardo Martinez-Valdes[1]

Email: e.a.martinezvaldes@bham.ac.uk

## Author Affiliations

[1]Centre of Precision Rehabilitation for Spinal Pain (CPR Spine), School of Sport, Exercise and Rehabilitation Sciences, College of Life and Environmental Sciences, University of Birmingham, Birmingham, UK.

[2]Rehabilitation Research Laboratory 2rLab, Department of Business Economics, Health and Social Care, University of Applied Sciences and Arts of Southern Switzerland, Manno/Landquart, Switzerland.

Email: Ignacio Contreras-Hernandez[1] iac921@student.bham.ac.uk – Deborah Falla[1] d.falla@bham.ac.uk – Alessandro Schneebeli[1 2] alessandro.schneebeli@supsi.ch – Eduardo Martinez-Valdes[1] e.a.martinezvaldes@bham.ac.uk

**WORD COUNT MAIN TEXT: 3865 (excluding title page, abstract, references, figures and tables)**

For peer review only

# Abstract

**Introduction:** Achilles tendinopathy is a debilitating overuse injury characterised by pain, impaired functional performance, morpho-mechanical changes to the Achilles tendon, and triceps surae neuromuscular alterations. Loading-based exercise has become the principal nonsurgical choice for the treatment of Achilles tendinopathy, however, mechanistic evidence by which loading-based treatment may help to resolve tendon pain remains unclear. This systematic review aims to summarize the evidence of the neuromechanical changes produced by Achilles tendinopathy and by exercise-induced mechanical loading.

**Methods and analysis:** This systematic review protocol was informed and reported in accordance with the Preferred Reporting Items for Systematic Review and Meta-Analysis (PRISMA-P) and the Cochrane Handbook for Systematic Reviews of Interventions. Pubmed, MEDLINE, EMBASE, CINAHL Plus, Web of Science, and SPORTDiscus electronic databases will be searched from inception to February 2021. Additionally, grey literature and key journals will be reviewed. Risk of bias will be determined independently by two reviewers using RoB2 and ROBINS-I tools according to Cochrane recommendations. Quality of the cumulative evidence will be assessed with the Grading of Recommendations, Assessment, Development, and Evaluation (GRADE) guidelines. If homogeneity exists between groups of studies, a random-effects meta-analysis will be conducted. If not, results will be synthesised narratively.

**Ethics and dissemination:** Our findings will be disseminated through publication in a peer-review journal and presented at conferences. No ethical approval was required.

**Prospero registration number:** CRD42021231933


**Keywords:** Achilles tendinopathy; neuromechanical properties, neuromuscular features, mechanical loading.

**WORD COUNT: 231**

For peer review only

# Article Summary

## Strengths and limitations of this study

- This will be the first systematic review to synthesise evidence of neuromechanical changes produced by Achilles tendinopathy.
- This will also be the first systematic review examining the effect of exercise-induced mechanical loading protocols on neuromechanical changes observed in individuals with Achilles tendinopathy.
- In accordance with the Grading of Recommendations, Assessment, Development, and Evaluation framework, the inclusion of observational studies might reduce the quality of evidence.

For peer review only

# INTRODUCTION

Tendinopathy describes a spectrum of pathological changes to the tendon, leading to pain and reduced function. The essence of tendinopathy is a failed healing response, characterised by abnormalities in the microstructure, composition, and cellularity of the tendon[1], with degeneration and disorganized proliferation of tenocytes, disruption of collagen fibres, and subsequent increase in non-collagenous matrix.[2]

Achilles tendinopathy (AT) is a debilitating overuse injury which causes considerable morbidity and functional impairment among the athletic and general population.[3 4] Achilles tendon injuries can be separated into non-insertional tendinopathy (55%-65% of the injuries), insertional tendinopathy (20%-25%), and proximal musculotendinous junction injuries (9%-25%), according to the location of pain[5]. Nevertheless, individuals may present symptoms at the insertion and mid-portion concurrently, and approximately 30% have bilateral pain[6]. Morphological comparisons of tendinopathic and healthy tendons have demonstrated a larger cross-sectional area (CSA) for the degenerated Achilles tendon.[7] It is believed that this increase is due to an accumulation of water and increased ground substance as a result of the pathology.[8] In addition, changes to the tendon's stiffness has been reported.[7] Typically, a thicker tendon is considered mechanically stronger due to its ability to dissipate high stresses (force/area) across the tendon and yield lower strain energy.[7] However, degenerated Achilles tendon has lower stiffness and Young's modulus compared with healthy tendons.[7]

Human movement emerges from the interplay among descending output from the central nervous system (CNS), sensory input from the body and environment, muscle dynamics and the whole body dynamics.[9] Thus, neuromechanics is the study of the coupling between neural information processing and mechanical behavior[9]. Fundamentally, the CNS plans, initiates and sends motor commands to muscle[10] then, the muscle executes the motor command producing force to pull the tendon, and finally, the tendon transmits and modulates muscle force controlling the movement[11]. Additionally, peripheral components of this hierarchical system (i.e., muscle spindles and Golgi tendon organs) send feedback signals to assist the CNS in motor commands planning[12].

For peer review only

Tendons require the ability to withstand, store, and then deliver substantial force to perform day-to-day activities.[13] During sports-related activities, where the repetition and speed of the loading are drastically increased, the mechanical force placed on the tendon becomes substantially amplified.[14] Therefore, a decrease in tendon stiffness will cause the muscle fascicles to shorten more to account for the increased compliance of the tendon.[7] This may limit the muscle's ability to function within the force-length curve's optimal region, thereby affecting movement economy.[7]

In addition to the morpho-mechanical changes induced by AT, several neuromechanical adaptations have been reported in individuals with AT.[11] Neuromechanical adaptations include how the mechanical system may offload a task that the neural system needs to accomplish[9] and it is possible only through a tight connection between sensory and motor systems. One of these neuromechanical adaptations is the electromechanical delay (EMD), which is defined as the time lag between muscle activation and the mechanical force produced, which dictates the muscle-tendon unit's temporal efficiency.[11] Longer EMD has recently been reported for the affected side compared with the non-affected side in individuals with Achilles tendinosis, indicating a compromised triceps surae musculotendinous unit temporal efficiency (ability to transmit force from the muscle to the tendon as quickly as possible).[11]

Other neuromechanical adaptations are the evoked spinal reflexes assessed by Ia-afferent-mediated H-reflex and the net excitation of the neuron pool determined by the first volitional wave (V-wave)[15]. Higher V-wave but not H-reflex values of the soleus muscle have been observed in the affected legs of athletes with chronic middle-portion Achilles tendinopathy[16]. This greater V-wave may result from an enhanced neural drive in the descending corticospinal pathways, elevated net excitability of both large and small motor neurons, and/or alterations in the presynaptic inhibition during voluntary activation of the soleus muscle[17]. However, a recent study using a similar approach has shown higher H-reflex and V-wave values in individuals with mid-portion AT compared with controls[11], showing that there is some discrepancy across studies.

Once symptoms develop, ensuing movement dysfunction may contribute to the chronicity of symptoms.[18] Pain in the Achilles tendon causes widespread motor inhibition in the affected region, evidenced by the lower electrical activity of the agonist, synergist, and antagonist

For peer review only

muscles.[19]. Individuals with tendinopathy also tend to use movement patterns that place excessive or abnormal load on their tendons; the faulty movement may represent either a root cause or a reason for chronicity or slow resolution.[18] This may be attributed to a protective mechanism that prevents further injury or even tendon rupture.[20]

Research on the treatment of AT is somewhat scarce despite the prevalence.[21] Over the past decade, loading-based treatment in the form of eccentric training (exercises where tendon lengthening during active contractions is emphasized) has become the main non-surgical choice of treatment for AT,[22] although there is no convincing evidence showing that this form of exercise is the most effective for AT. A recent systematic review concluded that there is little clinical or mechanistic evidence supporting the use of isolated eccentric exercises alone.[23] Well-conducted studies comparing different loading programs are largely lacking.[23] The purpose of exercise is to provide mechanical loading to the tendon in order to promote remodeling, decrease pain, and improve calf-muscle endurance and strength.[6 24] It seems that loading itself yields positive clinical, structural, and biochemical effects with respect to tendinopathy.[6 25-28] However, the successful management of AT remains challenging, possibly due to a lack of knowledge about the effect of loading parameters i.e., load progression, load magnitude, frequency (sets and repetitions), and restitution between treatment sessions.[21]

There are several systematic reviews about the effects of exercise in individuals with AT. However, most are focused on pain and function and usually only use self-reported outcome measures as a main outcome. Self-reported outcomes have high variability among the population, thus, the conclusion of these studies may be partially biased. Although two recent systematic reviews explored the effects of exercise on the morphological properties of the Achilles tendon in individuals with mid-portion AT,[29 30] to our knowledge, there are currently no systematic reviews examining the neuromechanical changes that occur in individuals with AT or the effects of exercise on these properties. Therefore, the aim of this systematic review is to synthesise the current literature regarding 1) Triceps surae-Achilles tendon complex neuromechanics in individuals with AT and 2) The effect of exercise-induced mechanical tendon loading on neuromechanical changes induced by AT.

For peer review only

# METHODS

This systematic review protocol has been developed following the Cochrane Handbook for Systematic Reviews of Interventions and the Preferred Reporting Items for Systematic Review and Meta-analysis Protocols (PRISMA-P) 2015 checklist (supplementary file 1).[31-33] This protocol has been registered with the International Prospective Register of Systematic Reviews (PROSPERO) on 26-01-2021 (Registration number: CRD42021231933). The Cochrane Handbook mainly focuses on the synthesis of evidence from intervention studies which is related only to the second objective of this systematic review, unfortunately, the first objective is commonly addressed by observational studies. Therefore, we followed these guidelines in addition to the COSMOS-E guidance for the following reasons: First, there are many similarities in the general structure and procedures used in both types of reviews. Second, the information in the Cochrane Handbook is described in more detail and includes important information related to observational studies. Third, a similar approach has been made in other systematic review protocols.[34 35] Fourth, widely accepted standards of systematic reviews meta-analysis of observational studies are lacking.[36] We know that some methodological differences exist, but we will pay particular attention to certain steps of the conducted process (e.g., choice of statistical methods, sources of heterogeneity, etc.)[36]

## Eligibility criteria

The PICOS framework (Population, Intervention, Comparison, Outcomes, and Study Design) has been used to define the eligibility criteria for the inclusion and exclusion of studies in this systematic review.[32 37] However, due to the characteristics of this research, we will use "Indicator" in the same category of "Intervention" as it has been used previously.[34 35]

## Population

The population of interest is adults (aged 18 to 65 years) with mid-portion or insertional Achilles tendinopathy and pain-free adults as a control group. However, we will also include studies that have used the asymptomatic lower limb as a control. In order to avoid excluding relevant articles identified during the scoping search, participants with bilateral Achilles

For peer review only - http://bmjopen.bmj.com/site/about/guidelines.xhtml

tendinopathy will also be included. There will be no restrictions in terms of gender or ethnicity. Studies that include individuals with Achilles tendinopathy who have been diagnosed with an underlying medical pathology or disorder (e.g. systemic inflammatory conditions, cardiovascular diseases, neurological disorders) and/or history of Achilles tendon surgery will be excluded.

**Intervention/Indicator**

In order to address the first aim of this systematic review, eligible studies will be those which include the use of any electrophysiological technique (e.g., surface electromyography, intramuscular electromyography, high-density surface electromyography, transcranial magnetic stimulation, cervicomedullary magnetic stimulation, or peripheral nerve stimulation) to determine neuromuscular, spinal, subcortical, or supraspinal changes in people with AT. Additionally, eligible studies will be those which include the use of ultrasonographic or Magnetic Resonance Imaging (MRI) to measure the morphological or mechanical properties of the Achilles tendon in people with AT. Similarly, eligible studies to address the second aim of this systematic review will be those which include the use of any electrophysiological technique to determine neuromuscular, spinal, subcortical, or supraspinal changes produced by any exercise-induced mechanical tendon loading protocols (e.g., eccentric, isometric, or concentric contractions, plyometric exercises, stretching or rehabilitation protocols) in people with AT. Moreover, eligible studies will be those which include the use of ultrasonographic or MRI to measure changes in the morphological or mechanical properties of the Achilles tendon produced by any exercise-induced mechanical loading in individuals with AT.

**Comparison**

Studies must include a comparison of the neuromuscular properties of the triceps surae muscle or morpho-mechanical properties of the Achilles tendon between individuals with AT and controls or between symptomatic and asymptomatic lower limbs. Likewise, studies assessing the effects of exercise-induced mechanical tendon loading should include a comparison of the neuromuscular features of the triceps surae muscle or morpho-mechanical properties of the Achilles tendon in individuals with AT and controls or between symptomatic and asymptomatic lower limbs.

For peer review only

## Outcomes

Primary outcomes will include neuromuscular properties of the triceps surae muscle and morpho-mechanical features of the Achilles tendon. We will include studies assessing the amplitude and timing of EMG activity of the gastrocnemius-soleus (millivolts and milliseconds, respectively), tibialis anterior and gastrocnemius-soleus co-activation (co-contraction ratio, %), gastrocnemius-soleus motor evoked potentials (MEPs, microvolts) obtained from transcranial and cervicomedullary magnetic stimulation (TMS and CMEPs), H-reflex (peak-to-peak amplitude, microvolts), F-wave or V-wave obtained by peripheral nerve stimulation (tibial nerve) and motor unit data (motor unit discharge rate, Hz) obtained from intramuscular and/or high-density surface EMG recordings (millivolts). The present systematic review will also include morpho-mechanical properties such as length (cm), thickness (mm), cross-sectional area ($mm^2$), volume ($cm^3$), stiffness (N/mm or kPa), modulus (kPa), creep, elasticity (kPa), strain (%), and stress (kPa) of the Achilles tendon. Only studies that measure any of these neuromechanical properties quantitatively will be included. Secondary outcomes will include duration of symptoms (months), the severity of symptoms (Victorian Institute of Sports Assessment-Achilles questionnaire (VISA-A) or Visual Analog Scale (VAS)), type of tendinopathy (mid-portion, insertional or both), diagnostic confirmation (clinical assessment and/or ultrasound evaluation), and physical activity level (International Physical Activity Questionnaire (IPAQ), hours of physical activity per week, etc.).

## Study design

Based on scoping searches, randomised controlled trials and non-randomised controlled trials (i.e., cohort, cross-sectional, and cohort studies) will be considered to address both objectives of this systematic review adequately. Non-original literature (e.g., systematic and narrative reviews) or other types of studies will be excluded and reported in the PRISMA flow diagram.

## Information sources

The following electronic databases will be used from inception to February 2021: Pubmed, MEDLINE (Ovid Interface), EMBASE (Ovid Interface), CINAHL Plus (EBSCO Interface), Web of Science (WOS; Clarivate Analytics) and SPORTDiscus (EBSCO Interface). Specific research strategies have been designed considering medical subject heading (MESH) terms to improve

For peer review only

search results. Moreover, hand searching of key journals will be conducted, including Journal of Applied Physiology, Journal of Orthopaedic & Sports Physical Therapy, Journal of Electrophysiology and Kinesiology, Journal of Biomechanics, Clinical Biomechanics, British Journal of Sports Medicine, Medicine and Science in Sports and Exercise, Journal of Science and Medicine in Sports, and Isokinetic and Exercise Science. The eligibility of the manuscripts found in hand searching will be defined using the PICOS framework. Additionally, relevant authors in the field will be contacted to identify unpublished articles in preparation. To minimise risk of bias publication, grey literature will be also included, and searches will be conducted using the British national bibliography for report literature (BNBRL), ProQuest Dissertations & These Global, OpenGrey database and EThOs. Reference lists of included studies and relevant systematic reviews will be checked for any further studies, accordingly with the MECIR standards.[38]

**Search strategy**

Two independent reviewers (IC and AS) will complete the search and identify potential studies to be included in this systematic review. There will be no restrictions in terms of date, design, or language, to ensure inclusion of all relevant articles.

Due to the inability to obtain maximal retrieval of articles during the scoping search and to adequately address both objectives of this systematic review, this search will be conducted in a two steps process:

1.- Initial search to identify studies with neuromuscular properties of the triceps surae or morpho-mechanical features of the Achilles tendon in individuals with AT.

2.- Secondary search identifying studies assessing the effects of exercise-induced tendon mechanical loading on neuromuscular properties of the triceps surae or the morpho-mechanical characteristics of the Achilles tendon in individuals with AT.

A search strategy example for MEDLINE (Ovid Interface) database of each step is reported in online supplementary file 2 and includes MESH terms, keywords and search strings to ensure maximal retrieval.[39] The specific search terms will be modified to reflect differences in keywords and syntax between databases but search strategy consistency will be guaranteed.

For peer review only

**Data management**

Literature search results, including citation and abstract of potentially eligible studies, will be imported into EndNote X9 (Clarivate Analytics PCL) reference manage software by one reviewer (IC), allowing the identification and removal of any duplicates before the screening process. Abstracts and full-texts of potentially eligible studies will be saved in an individual folder for each reviewer (IC, AS) and eligible studies will be retrieved and stored in EndNote X9. To effectively accomplish the screening process, forms that have been developed to reflect the inclusion and exclusion criteria will be used.

**Study selection**

Before the screening process, screening forms will be tested by two reviewers (IC, AS) in a small number of articles to ensure their effectiveness. The screening process will then start with the assessment of titles and abstracts of identified studies by two reviewers (IC, AS), and they will subcategorise them into definitely eligible, definitely ineligible, or doubtful.[40] In the event of disagreement, reviewers will first attempt to resolve through discussion; however, if consensus cannot be reached, a third reviewer (EMV) will mediate the process. Then, the reviewers will perform full-text screening of potentially eligible studies independently. Similarly, if no consensus is possible, a third reviewer will support the process. The agreement between the reviewers during both screening stages will be determined using the kappa statistic, and the PRISMA flow diagram will be used to summarise the study selection process.[32]

**Data collection process**

The data collection process will begin developing a standardised form based on the Cochrane data extraction template, objectives of the systematic review, and inclusion criteria as a guide. A standardised form will be piloted a priori on a sub-group of studies. IC will extract data, and AS will check the accuracy of this process. Any discrepancies will be discussed between the two reviewers; however, the third reviewer (EMV) will determine which data are relevant if no agreement is achieved. Authors of the primary studies will be contacted if any critical information that needs to be extracted is missing. If multiple publications of the same study exist, they will be collated, the primary authors contacted for further clarification and the duplicates removed. Likewise, if potentially eligible studies appear to use the same data during the data collection

For peer review only

process, the primary authors will be contacted, and a specific report will be selected. The decision of the selected report will be justified.

## Data items

Data items to be extracted include general study information, participants´ characteristics, measurement methods, and outcome measures. These items are presented in Table 1. We will use the same extraction sheet for each step of the systematic review; the only difference will be the item regarding "Type of exercise-induced mechanical tendon loading protocol applied". If any eligible studies include more than two groups, data will be extracted only from the control group and the one that meets the eligibility criteria.

**Table 1.** Characteristics of included studies

| **Date of data extraction:** | |
|---|---|
| **Information about data** | **Data extracted** |
| General study information | Title<br>Authors<br>Year of publication |
| Study Methodology | Study design<br>Sample size<br>Individuals' characteristics (age, gender, weight, height, physical activity level, etc)<br>Diagnostic confirmation (clinical evaluation, ultrasound/MRI assessment, use of questionnaires, etc)<br>Achilles tendinopathy group characteristics (location, side, pain intensity, duration of symptoms, etc)<br>Type of instrument used to measure the neuromuscular properties<br>Type of instrument used to determine the morpho-mechanical properties (Ultrasonography or MRI)<br>Type of exercise-induced mechanical tendon loading protocol applied |
| Outcome | The neuromuscular properties include the amplitude and timing of EMG activity of the gastrocnemius-soleus, tibialis anterior and gastrocnemius-soleus co-activation, gastrocnemius-soleus MEPs, H-reflex, F-wave, and motor unit data.<br>The morpho-mechanical properties of the Achilles tendon include length, thickness, cross-sectional area, volume, stiffness, modulus, creep, elasticity, strain, and stress. |

For peer review only - http://bmjopen.bmj.com/site/about/guidelines.xhtml

| | |
|---|---|
| | The comparison could be within-groups (e.g., affected versus non-affected side) or between-groups (e.g., Achilles tendinopathy group versus control group). |
| Funding, declaration of conflict of interest | Funding information<br>Conflict of interest of authors |

**Risk of bias**

The risk of bias will be determined independently by two reviewers (ICH and AS) using the RoB 2 and ROBINS-I tools according with Cochrane recommendations.[31] The RoB 2 is a tool to determine the risk of bias in randomised trials and includes the following domains: bias arising from the randomisation process, bias due to deviations from intended interventions, bias due to missing outcome data, bias in measurement of the outcome, bias in selection of the reported result, and overall bias.[41] The domains were selected to address all important mechanisms by which bias can be introduced into the results of a trial, based on a combination of empirical evidence and theoretical considerations.[41] Each domain is required, and no additional domains should be added. For each domain, the tool comprises a series of "signalling questions", a judgement about risk of bias for the domain, free text boxes to justify responses to the signalling questions and risk-of-bias judgement, and an option to predict (and explain) the likely direction of bias.[31] Signalling questions aim to elicit information relevant to an assessment of risk of bias.[41] The questions seek to be reasonably factual in nature.[41] The response options are "yes," "probably yes," "probably no," "no," and "no information".[41] Bases on these responses, the options for a domain-level risk-of-bias judgement are "Low", "Some concerns", and "High" risk of bias.[41]

The ROBINS-I will be used to determine the risk of bias in non-randomised studies of interventions and include the following domains: bias due to confounding, bias in selection of participants into the study, bias in classification of interventions, bias due to deviations from intended interventions, bias due to missing data, bias in measurement of outcomes, and bias in the selection of the reported result.[42] Each domain is mandatory, and no additional domain should be added. The tool comprises, for each domain a series of "signalling questions", a judgement about risk of bias for the domain, free text boxes to justify responses to the signalling questions and risk-of-bias judgements, and an option to predict (and explain) the likely direction of bias.[31] The signalling questions aim to elicit information relevant to the risk-of-bias judgement for the domain, and work in the same way as for RoB 2.[31] The response options are "yes," "probably yes,"

For peer review only

"probably no," "no," and "no information".[42] Bases on these responses, the options for a domain-level risk-of-bias judgement are "Low", "Moderate", "Serious" or "Critical" risk of bias, with an additional option of "No information".[42]

Disagreements between the reviewers regarding the risk of bias in a study will be resolved by discussion, with the involvement of a third review author (EMV) if necessary.

## Data synthesis

A meta-analysis will be considered if outcomes and methodology of the selected studies are homogenous. If possible, the two reviewers (IC, AS) will independently group studies that are more homogeneous using the following characteristics:

► Parameter used to determine neuromuscular properties of the Triceps Surae

► Parameter used to measure morpho-mechanical properties of the Achilles tendon

► Type of exercised-induced mechanical tendon loading protocol applied

Disagreement between the reviewers will be resolved by discussion, but if no agreement is possible, a third reviewer (EMV) will mediate the process.

Whether clinical or methodological homogeneity across studies investigating the same outcome domain is sufficient, statistical heterogeneity will be performed. The amount of inconsistency among studies will be assessed using the $I^2$ statistic.[43] As in previous reviews, the grouping of studies will be eligible for meta-analysis if an $I^2$ value of <50% (low heterogeneity) is determined.[44] Then, a random-effects meta-analysis will be performed for each subgroup following the recommendations of Deeks, et al, 2019.[45] Extracted data will be converted into a common rubric, which most likely be odds ratios with 95% confidence intervals since we are dealing mostly with binary data.[46] If the data are not sufficiently homogenous, the results of the considered outcomes will be described using the vote-counting procedure (direction of difference or no difference) and a narrative synthesis will be developed.[47] The narrative synthesis will be conducted following the recommendations of Popay, et al., 2006.[46]

## Confidence in cumulative evidence

Data pooled quality (certainty) will be assessed using the Grading of Recommendations Assessment, Development, and Evaluation (GRADE) approach. [48 49] This process include 5 steps described by Goldet and Howick, (2013)[50] and the final quality of evidence will be presented as 'High', 'Moderate', 'Low' or 'Very Low'. The certainty of evidence for each outcome across studies

can be decreased by risk of bias, inconsistency, indirectness, imprecision and publication bias.[51] Conversely, certainty of evidence can be increased by large effect size, dose-response gradient, and plausible confounding biases that underestimate the effect size.[51] Finally, recommendations for the interpretation of the evidence quality will be given, following the criteria of Guyatt et al., (2008).[52]

## Patient and public involvement

The topic of this systematic review protocol was not discussed at our established patient and public involvement meetings (PPI), due to COVID-19 pandemic. Patients will not be involved in the analysis and data collection of this project, but our results will be presented at PPI meetings at the University of Birmingham in the future.

## Ethics and dissemination of results

Ethical approval is not required for this review, as it will only involve the collation of previously published data. Our findings will be disseminated through publication in a peer-review journal and presented at national and/or international conferences.

**Author Contributions:** IC and EMV are responsible for the conception of the research question and development of the protocol. IC wrote the first draft of the protocol with guidance from EMV and DF. IC and AS will be the first and second reviewers, respectively. EMV will be the third reviewer. All drafts were revised and reviewed by all the authors before the final approval of the last version of the manuscript.

**Funding:** The authors have not declared a specific grant for this research from any funding agency in the public, commercial or not-for-profit sectors.

**Competing interests:** The authors have no competing interests to declare.

**Patients consent for publication:** Not required.

For peer review only

**Data availability statement:** This work is a systematic review protocol, and as such all data is derived from previously published work.

**Acknowledgements:** Not applicable.

For peer review only

## REFERENCES


1. Millar NL, Silbernagel KG, Thorborg K, et al. Tendinopathy. *Nature Reviews Disease Primers* 2021;7(1)
2. Longo UG, Ronga M, Maffulli N. Achilles tendinopathy. *Sports Med Arthrosc Rev* 2009;17(2):112-26. doi: 10.1097/JSA.0b013e3181a3d625 [published Online First: 2009/05/15]
3. Kvist M. Achilles tendon injuries in athletes. *Sports Med* 1994;18(3):173-201. doi: 10.2165/00007256-199418030-00004 [published Online First: 1994/09/01]
4. Rolf C, Movin T. Etiology, histopathology, and outcome of surgery in achillodynia. *Foot Ankle Int* 1997;18(9):565-9. doi: 10.1177/107110079701800906 [published Online First: 1997/10/06]
5. Kvist M. Achilles tendon injuries in athletes. *Ann Chir Gynaecol* 1991;80(2):188-201. [published Online First: 1991/01/01]
6. Silbernagel KG, Thomeé R, Eriksson BI, et al. Continued sports activity, using a pain-monitoring model, during rehabilitation in patients with Achilles tendinopathy: a randomized controlled study. *Am J Sports Med* 2007;35(6):897-906. doi: 10.1177/0363546506298279 [published Online First: 2007/02/20]
7. Arya S, Kulig K. Tendinopathy alters mechanical and material properties of the Achilles tendon. *J Appl Physiol (1985)* 2010;108(3):670-5. doi: 10.1152/japplphysiol.00259.2009 [published Online First: 2009/11/07]
8. Maffulli N, Moller HD, Evans CH. Tendon healing: can it be optimised? *Br J Sports Med* 2002;36(5):315-6. doi: 10.1136/bjsm.36.5.315 [published Online First: 2002/09/28]
9. Nishikawa K, Biewener AA, Aerts P, et al. Neuromechanics: an integrative approach for understanding motor control. *Integr Comp Biol* 2007;47(1):16-54.
10. Wolpert DM PKGC. The organization and planning of movement. In Principles of Neural Science. 5 ed. New York, NY: McGraw-Hill 2013.
11. Chang YJ, Kulig K. The neuromechanical adaptations to Achilles tendinosis. *J Physiol* 2015;593(15):3373-87. doi: 10.1113/jp270220 [published Online First: 2015/06/06]
12. Scott SH. Optimal feedback control and the neural basis of volitional motor control. *Nature Reviews Neuroscience* 2004;5(7):532-45. doi: 10.1038/nrn1427
13. Millar NL, Silbernagel KG, Thorborg K, et al. Tendinopathy. *Nat Rev Dis Primers* 2021;7(1):1. doi: 10.1038/s41572-020-00234-1 [published Online First: 2021/01/09]
14. Lin TW, Cardenas L, Soslowsky LJ. Biomechanics of tendon injury and repair. *Journal of Biomechanics* 2004;37(6):865-77. doi: https://doi.org/10.1016/j.jbiomech.2003.11.005
15. Knikou M. The H-reflex as a probe: pathways and pitfalls. *J Neurosci Methods* 2008;171(1):1-12. doi: 10.1016/j.jneumeth.2008.02.012 [published Online First: 2008/04/09]
16. Wang HK, Lin KH, Wu YK, et al. Evoked spinal reflexes and force development in elite athletes with middle-portion Achilles tendinopathy. *J Orthop Sports Phys Ther* 2011;41(10):785-94. doi: 10.2519/jospt.2011.3564 [published Online First: 2011/09/06]
17. Aagaard P, Simonsen EB, Andersen JL, et al. Neural adaptation to resistance training: changes in evoked V-wave and H-reflex responses. *J Appl Physiol (1985)* 2002;92(6):2309-18. doi: 10.1152/japplphysiol.01185.2001 [published Online First: 2002/05/17]
18. Scott A, Backman LJ, Speed C. Tendinopathy: Update on Pathophysiology. *J Orthop Sports Phys Ther* 2015;45(11):833-41. doi: 10.2519/jospt.2015.5884 [published Online First: 2015/09/22]
19. Henriksen M, Aaboe J, Graven-Nielsen T, et al. Motor responses to experimental Achilles tendon pain. *Br J Sports Med* 2011;45(5):393-8. doi: 10.1136/bjsm.2010.072561 [published Online First: 2010/06/15]


For peer review only

20. Lund JP, Donga R, Widmer CG, et al. The pain-adaptation model: a discussion of the relationship between chronic musculoskeletal pain and motor activity. *Can J Physiol Pharmacol* 1991;69(5):683-94. doi: 10.1139/y91-102 [published Online First: 1991/05/01]
21. Beyer R, Kongsgaard M, Hougs Kjær B, et al. Heavy Slow Resistance Versus Eccentric Training as Treatment for Achilles Tendinopathy: A Randomized Controlled Trial. *Am J Sports Med* 2015;43(7):1704-11. doi: 10.1177/0363546515584760 [published Online First: 2015/05/29]
22. Alfredson H, Pietilä T, Jonsson P, et al. Heavy-load eccentric calf muscle training for the treatment of chronic Achilles tendinosis. *Am J Sports Med* 1998;26(3):360-6. doi: 10.1177/03635465980260030301 [published Online First: 1998/06/09]
23. Malliaras P, Barton CJ, Reeves ND, et al. Achilles and patellar tendinopathy loading programmes : a systematic review comparing clinical outcomes and identifying potential mechanisms for effectiveness. *Sports Med* 2013;43(4):267-86. doi: 10.1007/s40279-013-0019-z [published Online First: 2013/03/16]
24. Silbernagel KG, Crossley KM. A Proposed Return-to-Sport Program for Patients With Midportion Achilles Tendinopathy: Rationale and Implementation. *J Orthop Sports Phys Ther* 2015;45(11):876-86. doi: 10.2519/jospt.2015.5885 [published Online First: 2015/09/22]
25. Alfredson H, Lorentzon R. Chronic Achilles tendinosis: recommendations for treatment and prevention. *Sports Med* 2000;29(2):135-46. doi: 10.2165/00007256-200029020-00005 [published Online First: 2000/03/04]
26. Kongsgaard M, Kovanen V, Aagaard P, et al. Corticosteroid injections, eccentric decline squat training and heavy slow resistance training in patellar tendinopathy. *Scand J Med Sci Sports* 2009;19(6):790-802. doi: 10.1111/j.1600-0838.2009.00949.x [published Online First: 2009/10/02]
27. Kongsgaard M, Qvortrup K, Larsen J, et al. Fibril morphology and tendon mechanical properties in patellar tendinopathy: effects of heavy slow resistance training. *Am J Sports Med* 2010;38(4):749-56. doi: 10.1177/0363546509350915 [published Online First: 2010/02/16]
28. Mafi N, Lorentzon R, Alfredson H. Superior short-term results with eccentric calf muscle training compared to concentric training in a randomized prospective multicenter study on patients with chronic Achilles tendinosis. *Knee Surg Sports Traumatol Arthrosc* 2001;9(1):42-7. doi: 10.1007/s001670000148 [published Online First: 2001/03/28]
29. Merza E, Pearson S, Lichtwark G, et al. Immediate and long-term effects of mechanical loading on Achilles tendon volume: A systematic review and meta-analysis. *Journal of Biomechanics* 2021:110289.
30. Färnqvist K, Pearson S, Malliaras P. Adaptation of Tendon Structure and Function in Tendinopathy With Exercise and Its Relationship to Clinical Outcome. *J Sport Rehabil* 2020;29(1):107-15. doi: 10.1123/jsr.2018-0353 [published Online First: 2019/03/13]
31. Higgins JPT TJ, Chandler J, et al. (editors). Cochrane Handbook for Systematic Reviews of Interventions version 6.0 (updated July 2019): Cochrane, 2019.
32. Shamseer L, Moher D, Clarke M, et al. Preferred reporting items for systematic review and meta-analysis protocols (PRISMA-P) 2015: elaboration and explanation. *BMJ : British Medical Journal* 2015;349:g7647.
33. Moher D, Shamseer L, Clarke M, et al. Preferred reporting items for systematic review and meta-analysis protocols (PRISMA-P) 2015 statement. *Systematic Reviews* 2015;4(1):1. doi: 10.1186/2046-4053-4-1
34. Arvanitidis M, Falla D, Sanderson A, et al. Does pain influence force steadiness? A protocol for a systematic review. *BMJ Open* 2021;11(1):e042525.

For peer review only

35. Devecchi V, Gallina A, Heneghan NR, et al. Are neuromuscular adaptations present in people with recurrent spinal pain during a period of remission? A protocol for a systematic review. *BMJ Open* 2019;9(12):e033276. doi: 10.1136/bmjopen-2019-033276 [published Online First: 2019/12/26]
36. Mueller M, D'Addario M, Egger M, et al. Methods to systematically review and meta-analyse observational studies: a systematic scoping review of recommendations. *BMC Med Res Methodol* 2018;18(1):44. doi: 10.1186/s12874-018-0495-9 [published Online First: 2018/05/23]
37. Smith V, Devane D, Begley CM, et al. Methodology in conducting a systematic review of systematic reviews of healthcare interventions. *BMC Medical Research Methodology* 2011;11(1):15.
38. Higgins JPT LT, Chandler J, Tovey D, Churchill R. Methodological Expectations of Cochrane Intervention Reviews. Cochrane: London, 2016.
39. Jenuwine ES, Floyd JA. Comparison of Medical Subject Headings and text-word searches in MEDLINE to retrieve studies on sleep in healthy individuals. *J Med Libr Assoc* 2004;92(3):349-53.
40. Lefebvre C GJ, Briscoe S, et al. Chapter 4: Searching for and selecting studies. In: Higgins JPT TJ, Chandler J, et al. , ed. Cochrane Handbook for Systematic Reviews of Interventions version 60 (updated July 2019): Cochrane, 2019.
41. Sterne JAC, Savović J, Page MJ, et al. RoB 2: a revised tool for assessing risk of bias in randomised trials. *BMJ* 2019;366:l4898. doi: 10.1136/bmj.l4898
42. Sterne JA, Hernán MA, Reeves BC, et al. ROBINS-I: a tool for assessing risk of bias in non-randomised studies of interventions. *BMJ* 2016;355:i4919. doi: 10.1136/bmj.i4919
43. Higgins JP, Thompson SG. Quantifying heterogeneity in a meta-analysis. *Stat Med* 2002;21(11):1539-58. doi: 10.1002/sim.1186 [published Online First: 2002/07/12]
44. Heneghan NR, Smith R, Rushton A. Thoracic dysfunction in whiplash-associated disorders: a systematic review and meta-analysis protocol. *Syst Rev* 2016;5:26. doi: 10.1186/s13643-016-0201-0 [published Online First: 2016/02/11]
45. Deeks JJ HJ, Altman DG. Chapter 10: analysing data and undertaking meta-analysis. In: Higgins JPT TJ, Chandler J, et al., ed. Cochrane Handbook for systematic reviews of interventions version 60 (updated July 2019): Cochrane, 2019.
46. Popay J RH, Snowden A. Guidance on the conduct of narrative synthesis in systematic reviews: a product from the ESRC methods programme. Lancaster, UK: Lancaster University, 2006.
47. McKenzie JE BS. Chapter 12: Synthesizing and presenting findings using other methods. In: Higgins JPT TJ, Chandler J,, ed. Cochrane Handbook for systematic reviews of interventions. London: Cochrane, 2008.
48. Guyatt G, Oxman AD, Akl EA, et al. GRADE guidelines: 1. Introduction-GRADE evidence profiles and summary of findings tables. *J Clin Epidemiol* 2011;64(4):383-94.
49. Schünemann H BJ, Guyatt G, eds. Handbook for grading the quality of evidence and the strength of recommendations using the GRADE approach2013.
50. Goldet G, Howick J. Understanding GRADE: an introduction. *J Evid Based Med* 2013;6(1):50-54.
51. Balshem H, Helfand M, Schünemann HJ, et al. GRADE guidelines: 3. Rating the quality of evidence. *J Clin Epidemiol* 2011;64(4):401-6.
52. Guyatt GH, Oxman AD, Vist GE, et al. GRADE: an emerging consensus on rating quality of evidence and strength of recommendations. *BMJ* 2008;336(7650):924-26.

For peer review only

**PRISMA-P (Preferred Reporting Items for Systematic Review and Meta-Analysis Protocols) 2015 checklist: recommended items to address in a systematic review protocol***

| Section and topic | Items N° | Checklist item | Page N° |
|---|---|---|---|
| **ADMINISTRATIVE INFORMATION** | | | |
| Title: | | | |
| Identification | 1a | Identify the report as a protocol of a systematic review | 1 |
| Update | 1b | If the protocol is for an update of a previous systematic review, identify as such | N/A |
| Registration | 2 | If registered, provide the name of the registry (such as PROSPERO) and registration number | 2,7 |
| Authors: | | | |
| Contact | 3a | Provide name, institutional affiliation, e-mail address of all protocol authors; provide physical mailing address of corresponding author. | 1 |
| Contributions | 3b | Describe contributions of protocol authors and identify the guarantor of the review | 15 |
| Amendments | 4 | If the protocol represents an amendment of a previously completed or published protocol, identify as such and list changes; otherwise, state plan for documenting important protocol amendments | N/A |
| Support: | | | |
| Sources | 5a | Indicate sources of financial or other support for the review | 15 |
| Sponsor | 5b | Provide name for the review funder and/or sponsor | N/A |
| Role of sponsor or funder | 5c | Describe role of funder(s), sponsor(s), and/or institution(s), if any, in developing the protocol | N/A |
| **INTRODUCTION** | | | |
| Rationale | 6 | Describe the rationale for the review in the context of what is already known | 4-6 |
| Objectives | 7 | Provide an explicit statement of the question(s) the review will address with reference to participants, interventions, comparators, and outcomes (PICO) | 6 |
| **METHODS** | | | |
| Eligibility criteria | 8 | Specify the study characteristics (such as PICO, study design, setting, time frame) and report characteristics (such as years considered, language, publication status) to be used as criteria for eligibility for the review | 7,8 |
| Information sources | 9 | Describe all intended information sources (such as electronic databases, contact with study authors, trial registers or other grey literature sources) with planned dates of coverage | 9,10 |
| Search strategy | 10 | Present draft of search strategy to be used for at least one electronic database, including planned limits, such that it could be repeated | Supplementary file 2 |

For peer review only

| | | | |
|---|---|---|---|
| Study records: | | | |
| Data management | 11a | Describe the mechanism(s) that will be used to manage records and data throughout the review | 11 |
| Selection process | 11b | State the process that will be used for selecting studies (such as two independents reviewers) through each phase of the review (that is, screening, eligibility, and inclusion in meta-analysis) | 11 |
| Data collection process | 11c | Describe planned methods of extracting data from reports (such as piloting forms, done independently, in duplicate) any processes for obtaining and confirming data from investigators | 11,12 |
| Data items | 12 | List and define all variables from which data will be sought (such as PICO items, funding sources), any-pre-planned data assumptions and simplifications | 11,12/Table 1 |
| Outcomes and prioritization | 13 | List and define all outcomes for which data will be sought, including prioritization of main and additional outcomes, with rationale | 11,12/Table 1 |
| Risk of bias in individual studies | 14 | Describe anticipated methods for assessing risk of bias of individual studies, including whether this will be done at the outcome or study level, or both; state how this information will be used in data synthesis | 13,14 |
| Data synthesis | 15a | Describe criteria under which study data will be quantitatively synthesised | 14 |
| | 15b | If data are appropriate for quantitative synthesis, describe planned summary measures, methods of handling data and methods of combining data from studies, including any planned exploration of consistency (such as $I^2$, Kendall's $\tau$) | 14 |
| | 15c | Describe any proposed additional analysis (such as sensitivity or subgroup analyses, meta-regression) | 14 |
| | 15d | If quantitative synthesis is not appropriate, describe the type of summary planned | 14 |
| Meta-bias(es) | 16 | Specify any planned assessment of meta-bias(es) (such as publication bias across studies, selective reporting within studies) | 14 |
| Confidence in cumulative evidence | 17 | Describe how the strength of the body of evidence will be assessed (such as GRADE) | 14,15 |

***It is strongly recommended that this checklist be read in conjunction with the PRISMA-P Explanation and Elaboration for important clarification on the items. Amendments to a review protocol should be tracked and dated. The copyright for PRISMA-P (including checklist) is held by the PRISMA-P Group and is distributed under a Creative Commons Attribution License 4.0.**

From: Shamseer L, Moher D, Clarke M, Ghersi D, Liberati A, Petticrew M, Shekelle P, Stewart L. PRISMA-P Group. Preferred reported items for systematic review and meta-analysis protocols (PRISMA-P) 2015: elaboration and explanations. BMJ. 2015 Jan 2; 349:g7647

For peer review only

## Search strategy step 1 MEDLINE

### Search string

("Corticospinal excita*" OR "cortical excita*" OR “spinal excita*” OR "spinal reflex" OR "H-reflex" OR "V-wave" OR "F-wave" OR "neural drive" OR "motor evoked potential*" OR "evoked muscle response*" OR "electromyogra*" OR "activation" OR "co-activation" OR "preactivation" OR "electromechanic*" OR "motor unit*" OR "motor neuron*" OR "length" OR "thickness" OR "cross sectional area" OR "modulus" OR "viscoelasticity" OR "elasticity" OR "strain" OR "stress" OR "stiffness" OR "volume" OR "creep" OR "mechanic*" OR "morphologic*") AND ("tendin*" OR "tenosynovitis" OR "paratenonitis" OR "tendovaginitis" OR "peritendinitis" OR "achillodynia") AND ("Achilles" OR "TendoAchilles" OR "Tendo-Achilles" OR "calcaneal tendon" OR "gastrocnemius" OR "triceps surae" OR "calf muscle*" OR "tibialis anterior")

### Keywords

1- Corticospinal excita*.mp
2- Cortical excita*.mp
3- Spinal excita*.mp
4- Spinal reflex.mp
5- H-reflex.mp
6- V-wave.mp
7- F-wave.mp
8- Neural drive.mp
9- Motor evoked potential*.mp
10- Evoked muscle response*.mp
11- Electromyogra*.mp
12- Activation.mp
13- Co-activation.mp
14- Preactivation.mp
15- Electromechanic*.mp
16- Motor unit*.mp
17- Motor neuron*.mp
18- Length.mp
19- Thickness.mp
20- Cross sectional area.mp
21- Modulus.mp
22- Viscoelasticity.mp
23- Elasticity.mp
24- Strain.mp
25- Stress.mp
26- Stiffness.mp
27- Volume.mp

For peer review only

28- Creep.mp
29- Mechanic*.mp
30- Morphologic*.mp
31- 1-30
32- Tendin*.mp
33- Tenosynovitis.mp
34- Paratenonitis.mp
35- Tendovaginitis.mp
36- Peritendinitis.mp
37- Achillodynia.mp
38- 32-37
39- Achilles.mp
40- TendoAchilles.mp
41- Tendo-Achilles.mp
42- Calcaneal tendon.mp
43- Gastrocnemius.mp
44- Triceps surae.mp
45- Calf muscle*.mp
46- Tibialis anterior.mp
47- 39-46
48- 31 AND 38 AND 47

For peer review only

## Search strategy step 2 MEDLINE

### Search string

("corticospinal excita*" OR "cortical excita*" OR “spinal excita*” OR "spinal reflex" OR "H-reflex" OR "V-wave" OR "F-wave" OR "neural drive" OR "motor evoked potential*" OR "evoked muscle response*" OR "electromyogra*" OR "activation" OR "co-activation" OR "preactivation" OR "electromechanic*" OR "motor unit*" OR "motor neuron*" OR "length" OR "thickness" OR "cross sectional area" OR "modulus" OR "viscoelasticity" OR "elasticity" OR "strain" OR "stress" OR "stiffness" OR "volume" OR "creep" OR "mechanic*" OR "morphologic*") AND ("mechanical load*" OR "Exercise*" OR "physical activity*" OR "eccentric" OR "concentric" OR "isometric" OR "training" OR "strengthening" OR "stretching" OR "vibration" OR "oscillation" OR "plyometric" OR "running" OR "walking") AND ("tendin*" OR "tenosynovitis" OR "paratenonitis" OR "tendovaginitis" OR "peritendinitis" OR "achillodynia") AND ("Achilles" OR "TendoAchilles" OR "Tendo-Achilles" OR "calcaneal tendon" OR "gastrocnemius" OR "triceps surae" OR "calf muscle*" OR "tibialis anterior")

### Keywords

1- Corticospinal excita*.mp
2- Cortical excita*.mp
3- Spinal excita*.mp
4- Spinal reflex.mp
5- H-reflex.mp
6- V-wave.mp
7- F-wave.mp
8- Neural drive.mp
9- Motor evoked potential*.mp
10- Evoked muscle response*.mp
11- Electromyogra*.mp
12- Activation.mp
13- Co-activation.mp
14- Preactivation.mp
15- Electromechanic*.mp
16- Motor unit*.mp
17- Motor neuron*.mp
18- Length.mp
19- Thickness.mp
20- Cross sectional area.mp
21- Modulus.mp
22- Viscoelasticity.mp
23- Elasticity.mp
24- Strain.mp

For peer review only

25- Stress.mp
26- Stiffness.mp
27- Volume.mp
28- Creep.mp
29- Mechanic*.mp
30- Morphologic*.mp
31- 1-30
32- Mechanical load*.mp
33- Exercise*.mp
34- Physical activity*.mp
35- Eccentric.mp
36- Concentric.mp
37- Isometric.mp
38- Training.mp
39- Strengthening.mp
40- Stretching.mp
41- Vibration.mp
42- Oscillation.mp
43- Plyometric.mp
44- Running.mp
45- Walking.mp
46- 32-45
47- Tendin*.mp
48- Tenosynovitis.mp
49- Paratenonitis.mp
50- Tendovaginitis.mp
51- Peritendinitis.mp
52- Achillodynia.mp
53- 47-52
54- Achilles.mp
55- TendoAchilles.mp
56- Tendo-Achilles.mp
57- Calcaneal tendon.mp
58- Gastrocnemius.mp
59- Triceps surae.mp
60- Calf muscle*.mp
61- Tibialis anterior.mp
62- 54-61
63- 31 AND 46 AND 53 AND 62

For peer review only
